# A Qualitative and Quantitative Analysis of a Pilot Psychotherapy Training and Simulation Workshop for a Cohort of Psychiatry Core Trainees Starting Their Long Case in SABP Foundation Trust

**DOI:** 10.1192/bjo.2024.295

**Published:** 2024-08-01

**Authors:** Amit Fulmali, Neelima Reddi

**Affiliations:** Surrey and Borders Partnership NHS Foundation Trust, Chertsey, United Kingdom

## Abstract

**Aims:**

Background: The uncertainty and anxieties about psychotherapy long case among trainees was high. This training was designed to alleviate stress and to increase knowledge and confidence among trainees.

The Primary aim of this project was to improve individual skills and awareness for Psychodynamic Psychotherapy. Secondary aim was to get feedback from trainees to improve future psychotherapy training.

The Null hypothesis (H_0_): There is no difference between the Pre and Post training questionnaire scores.

**Methods:**

This is a blinded study where the researcher cannot identify the participants. A mixed study approach is taken, with both quantitative and qualitative analysis used for this study. A pre training and post training questionnaire was provided to participants. This study collected quantitative data in the form of Likert scale questionnaire as a primary approach to test the hypothesis. The qualitative data was collected by open ended questions. The qualitative part is to understand the trainee's problems and what improvements have to be made in the workshop, to generate a structural model for effective practical psychotherapy training.

The sample consists of 13 Psychiatry Core Trainees at different levels of their training and 1 speciality doctor. 14 feedback questionnaires were available, 1 questionnaire was excluded as it did not fit the inclusion criteria.

**Results:**

The paired *t*-test was used for all the three quantitative questions: Knowledge of psychotherapy, Theoretical and Clinical Application of Psychotherapy. The t-test showed the difference between pre-and post-questionnaires scores to be statistically significant (p value < 0.05). So, we reject the Null Hypothesis. The effect size was large, with Cohen's d score > 0.8 for all three questionnaires.

Thematic analysis of Qualitative data was done. Codes were formed from the supporting quotes. Themes were derived from the similar codes. Four themes were created:

- Challenges experienced by core trainees.
- Emotions and confidence.
- Knowledge acquired.
- Suggestions for improvements.

**Conclusion:**

1. We can conclude that the Psychotherapy workshop was effective, and the Core Trainees have better insight than before.
2. The qualitative analysis results were in accordance with the quantitative analysis.
3. Challenges experiences by trainees in managing their own emotions were addressed in the training. Quote (IV) “it was very good! calmed nerves.”
4. There was increase in knowledge and confidence among trainees.
5. Suggestions were full day of training and to have more role playing; to demonstrate the psychological concepts like transference, countertransference, defences, resistances in role play.

